# Supplementary material for: Reduction of chromosomal instability and inflammation is a common aspect of adaptation to aneuploidy
Source: EMBO Rep. 2024 Sep 18;25(11):26. doi: 10.1038/s44319-024-00252-0 (PMC11549362; doi:10.1038/s44319-024-00252-0)
Supplement: Supplementary file 1 — Appendix [file 44319_2024_252_MOESM1_ESM.pdf]

Appendix

Reduction of chromosomal instability and inflammation is a common aspect of adaptation to aneuploidy

Hintzen et al.

Tabel of Contents

Appendix Figure S1.
Copy number analysis and correlation between proliferation and gene imbalances. P. 2-3

Appendix Figure S2:
Gene set enrichment for biological processes and individual clones P. 4-5

Appendix Figure S1: Copy number analysis and correlation between proliferation and gene imbalances

A

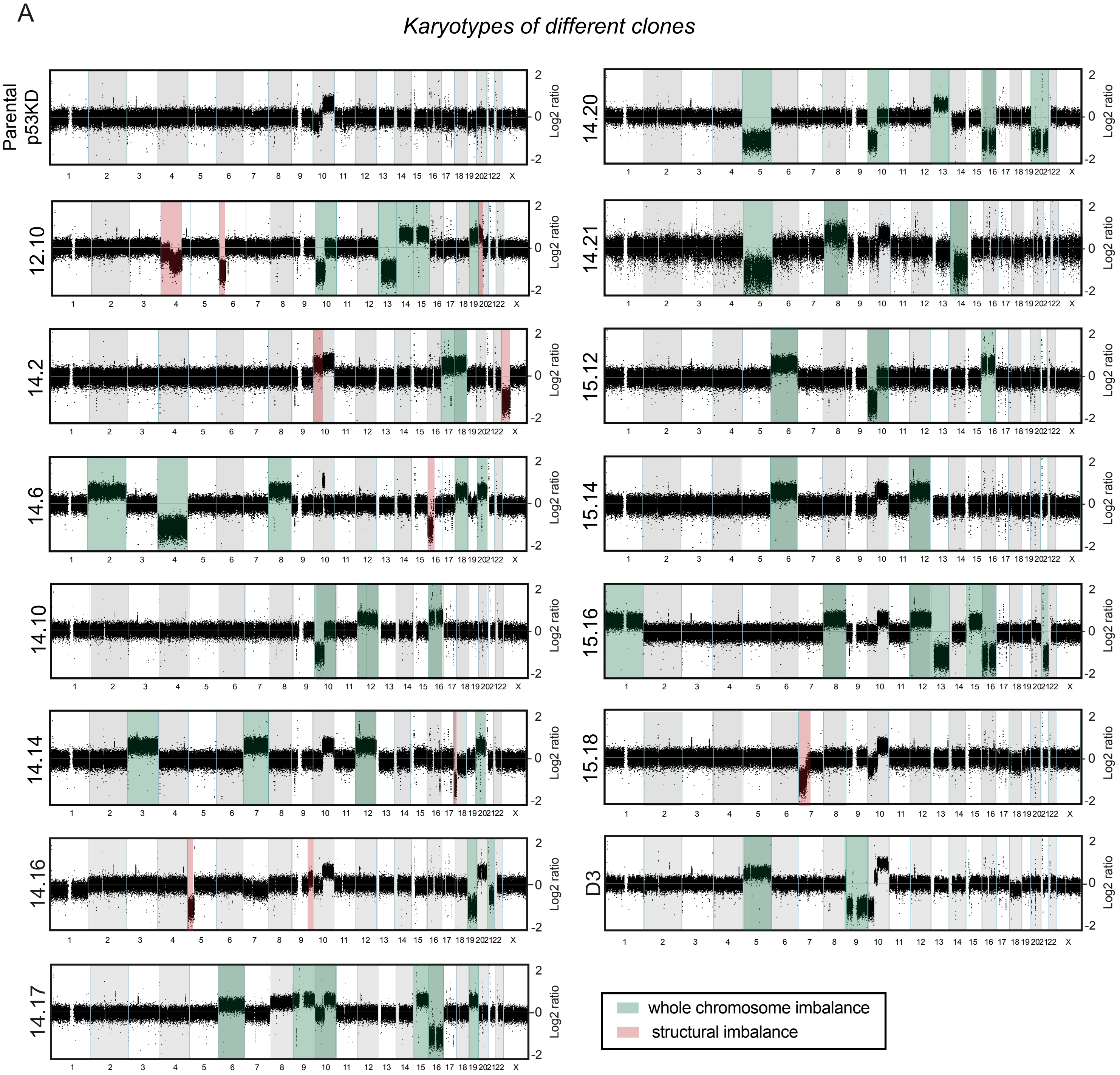

Appendix Figure S1: Copy number analysis and correlation between proliferation and gene imbalances
A. Genome-wide chromosome copy number profile as determined by low pass whole genome sequencing of the RPE-1 p53kd parental clone and 14 clones harboring chromosome imbalances. Chromosome gains and losses were depicted in green boxes (whole chromosomes) or red boxes (chromosome segments). Alterations of chromosome 10, already present in the parental cells, were not highlighted. B. (next page) Comparison between RNA and DNA sequencing for inferring karyotypes for clone 14.10, 14.16, 14.20 and 14.21; both early passage and adapted. Both RNA read counts and DNA read count were normalized over parental early. DNA copy number is displayed in Log2 ratio per bin and RNA sequencing are displayed as inferred copy number per bin. C (next page). Spearman correlation between RNA sequencing-derived gained and lost genes per clone and proliferation rates measured in Fig. 1B for early and adapted clones. Dots show mean, error bars indicate standard deviation, n= at least 2. Line shows linear regression model fitted to the data. Color-coding as determined in Fig. 2A.

Appendix Figure S1 continue:
Copy number analysis and correlation between proliferation and gene imbalances

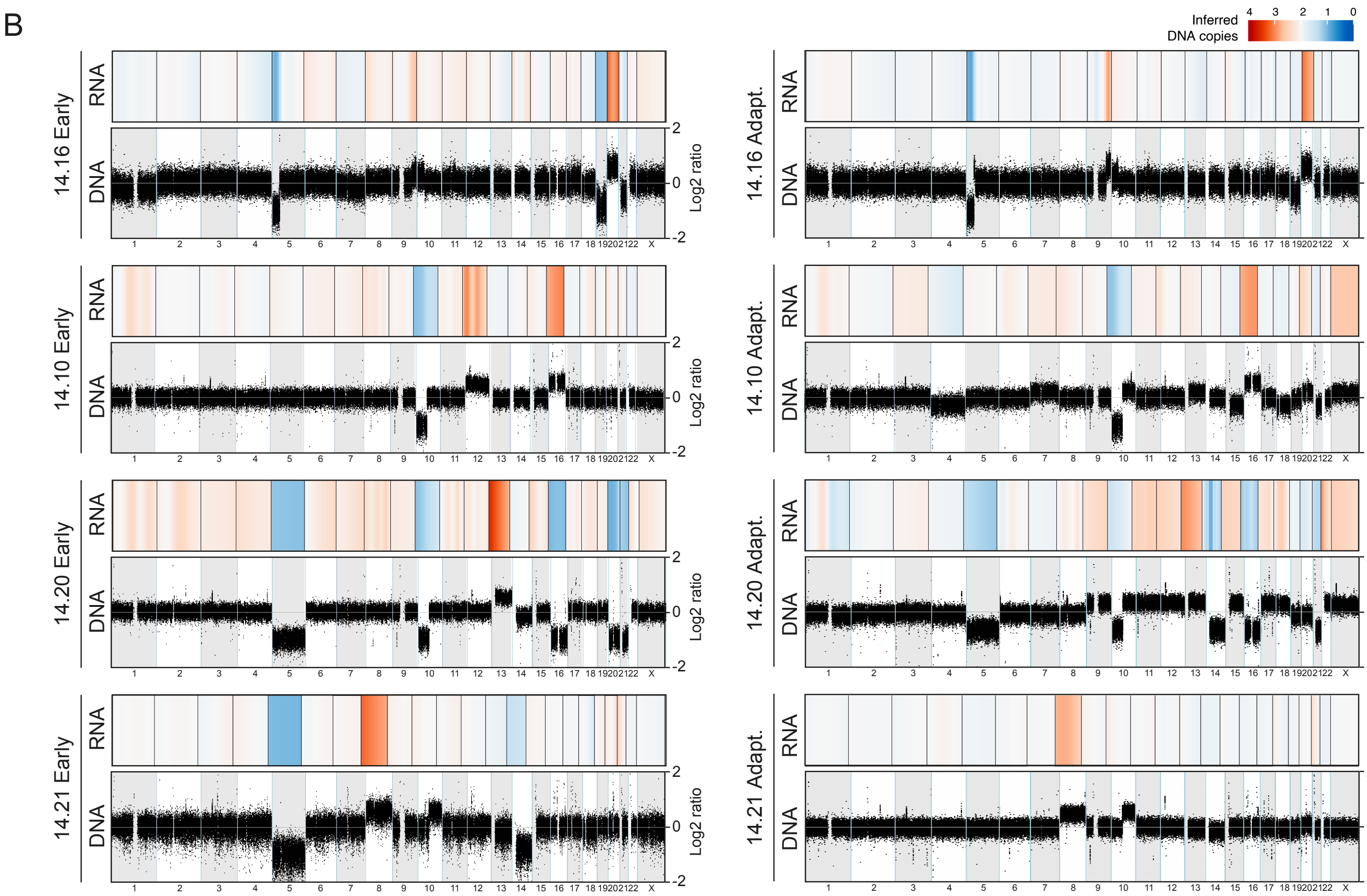

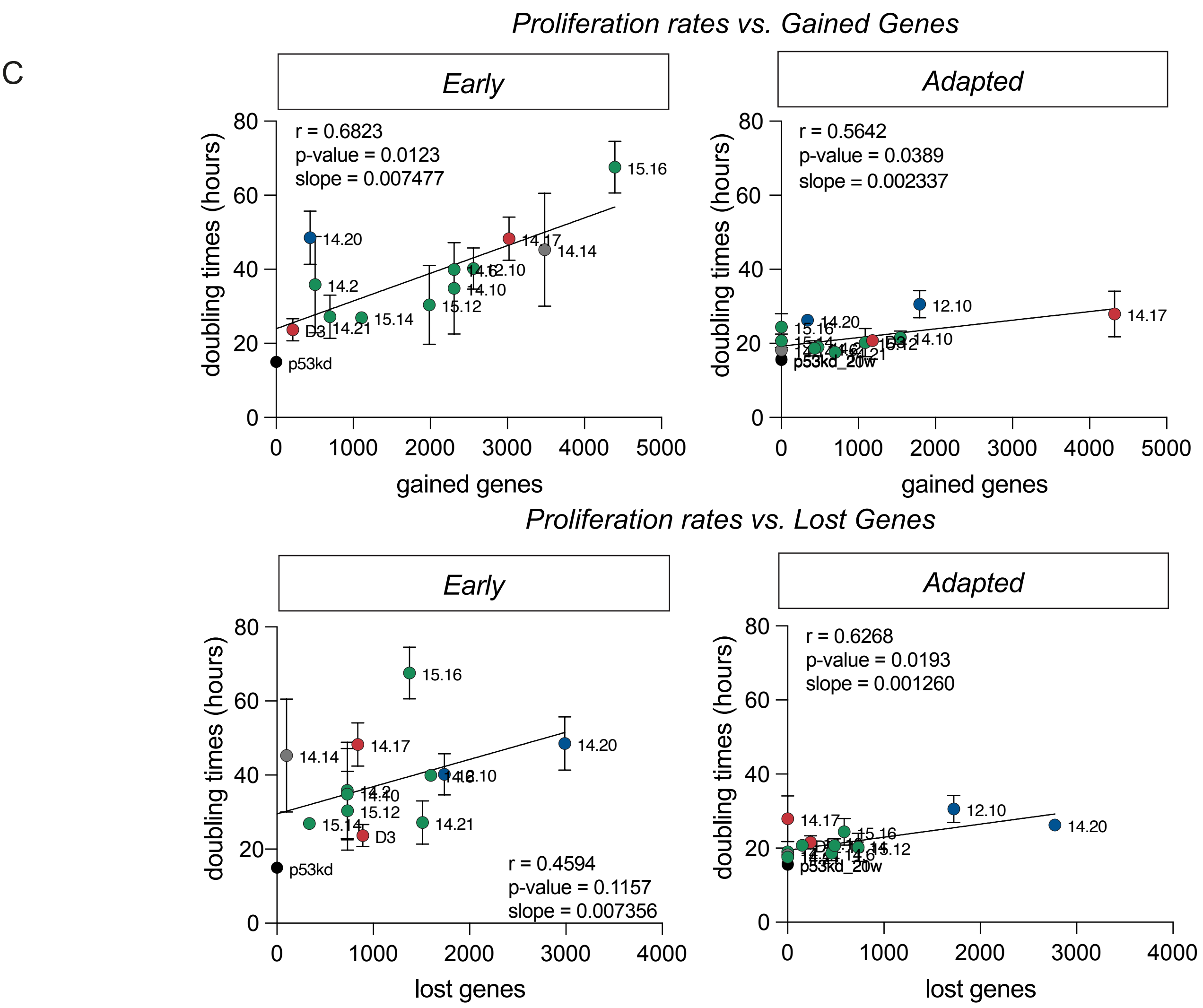

Appendix Figure S2: Gene set enrichment for biological processes and individual clones

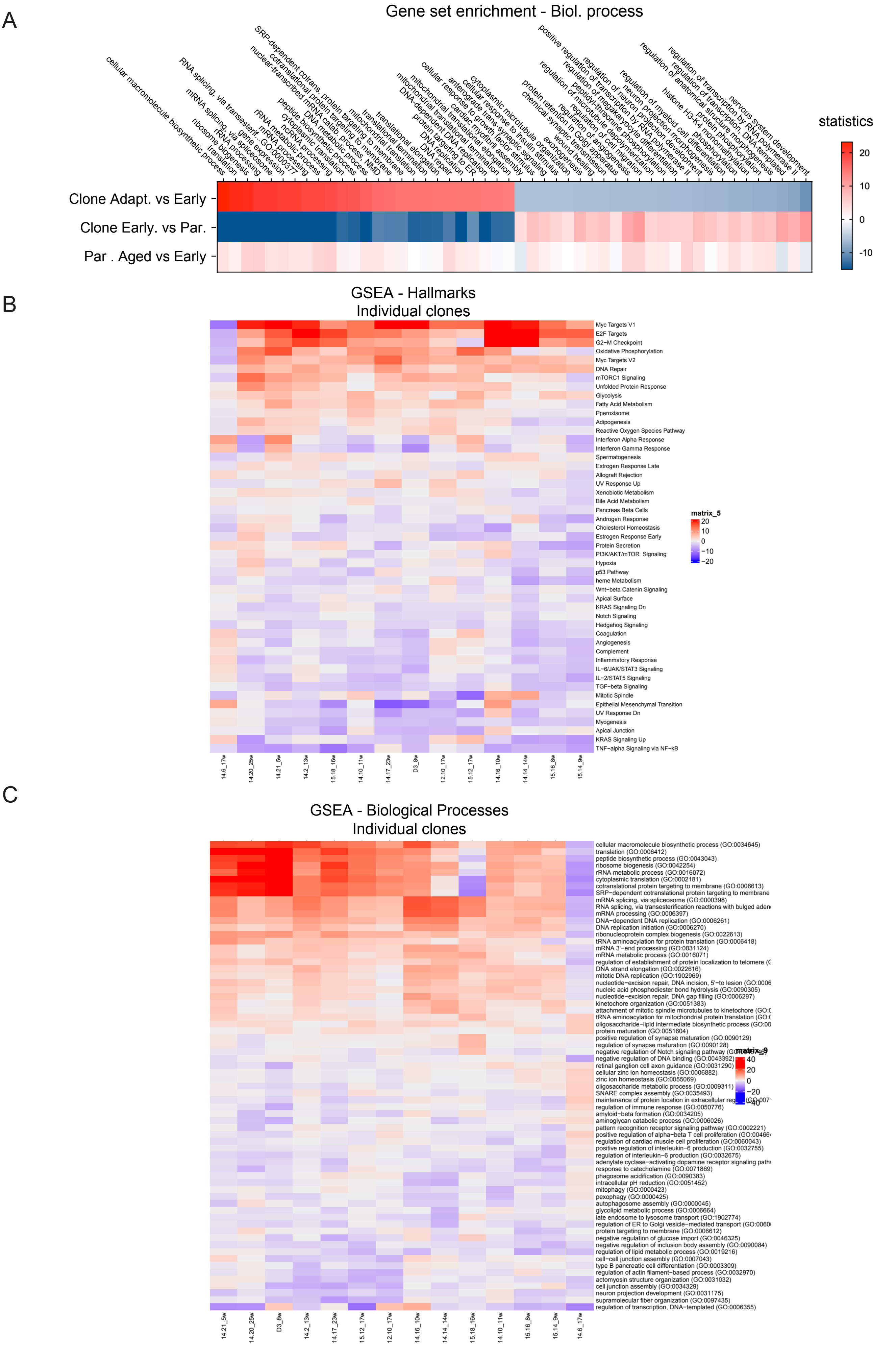

Appendix Figure S2 continue: Gene set enrichment for biological processes and individual clones

D

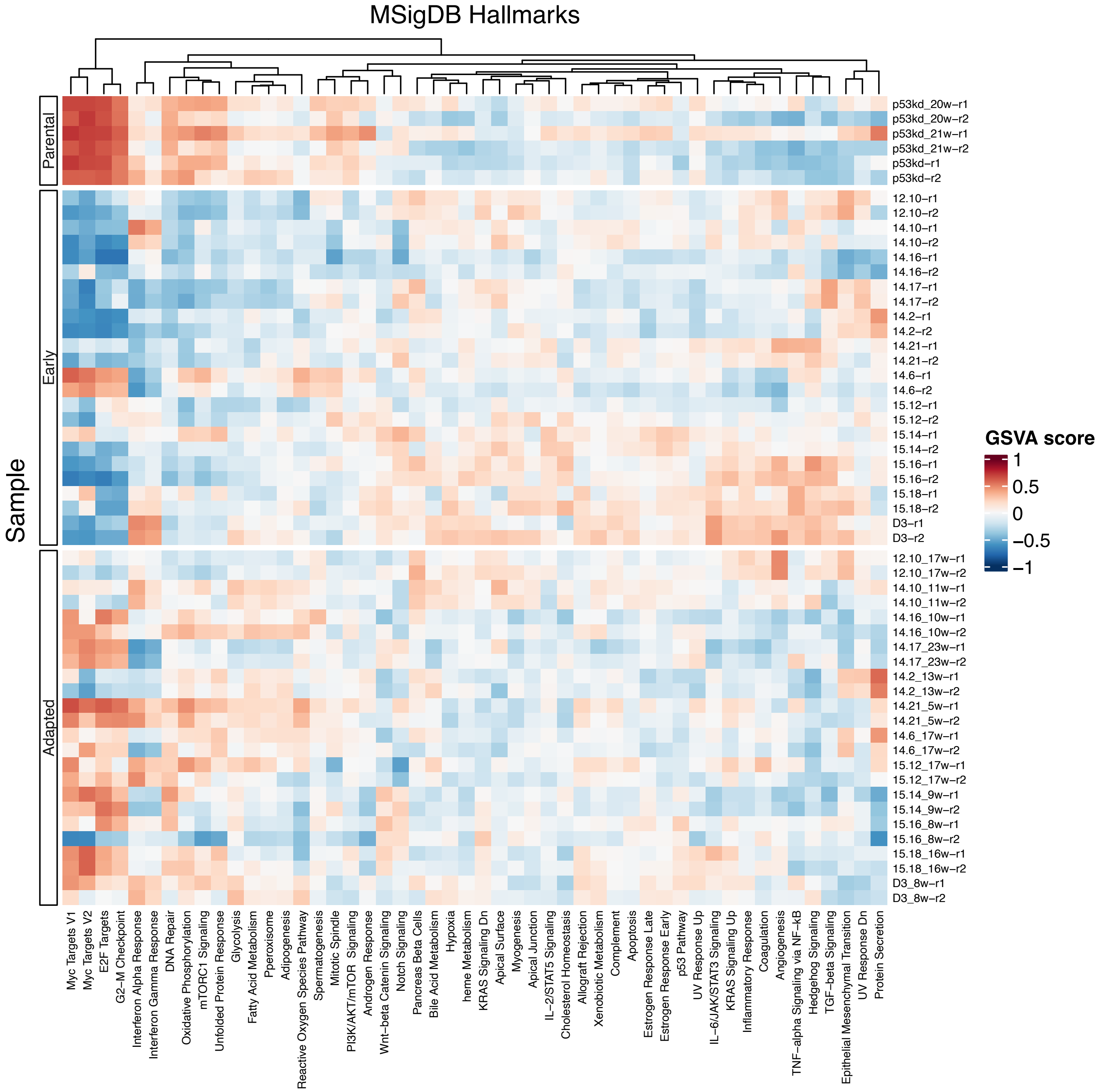

Appendix Figure S2: Gene set enrichment for biological processes and individual clones

A. (Previous page) Gene set enrichment analysis for GO Biological processes on the transcriptional alterations observed in all adapted clones compared to their early counterparts. The top 50 most altered gene sets are displayed (top row). Alterations in early clones over parental (middle row) and aged parentals (bottom row) are displayed as a reference. Colors indicate Wald statistics. B. Gene set enrichment analysis (GSEA) for the individual clones, evaluating up and downregulated GO hallmarks in adapted clones compared to their early counterparts. Two replicates of every clone were sequenced. Colors indicate Wald statistics. C. Gene set enrichment analysis (GSEA) for the individual clones, evaluating up and downregulated GO biological processes in adapted clones compared to their early counterparts. Two replicates of every clone were sequenced. Colors indicate Wald statistics. D. Heatmap showing Gene Set Variation Analysis (GSVA) for hallmarks calculated per clone per individual replicate. Colors indicate GSVA scores.
