# Supplementary figures and images for: Reduction of chromosomal instability and inflammation is a common aspect of adaptation to aneuploidy
Source: EMBO Rep. 2024 Sep 18;25(11):26. doi: 10.1038/s44319-024-00252-0 (PMC11549362; doi:10.1038/s44319-024-00252-0)

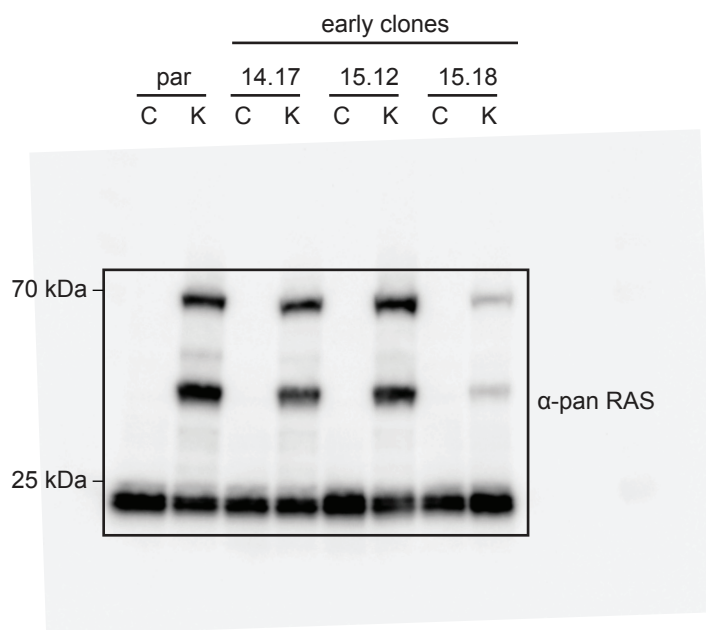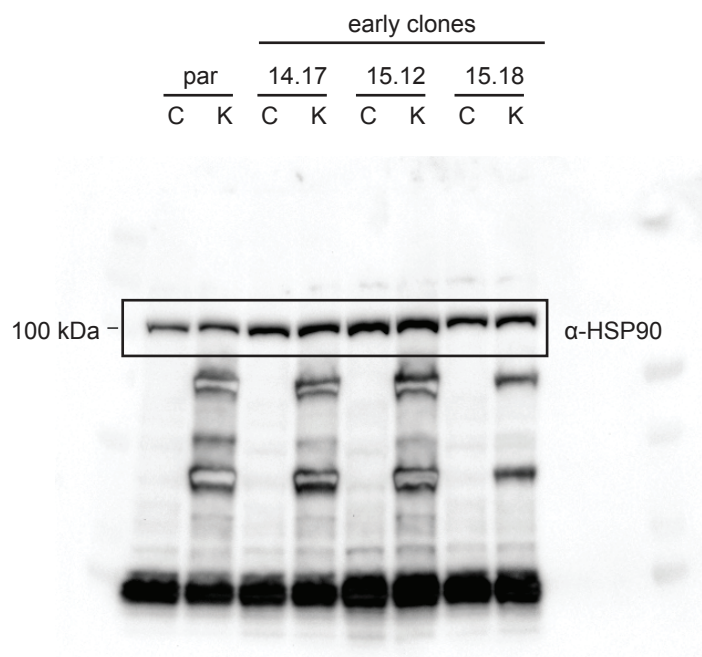

Supplement: Supplementary file 9 — Source data Fig. 6 [file 44319_2024_252_MOESM9_ESM.zip › Figure 6/Figure 6D image data- blot.pdf]
