# Supplementary material for: Reduction of chromosomal instability and inflammation is a common aspect of adaptation to aneuploidy
Source: EMBO Rep. 2024 Sep 18;25(11):26. doi: 10.1038/s44319-024-00252-0 (PMC11549362; doi:10.1038/s44319-024-00252-0)
Supplement: Supplementary file 10 — Expanded View Figures [file 44319_2024_252_MOESM10_ESM.pdf]

Expanded View Figures

Figure EV1. CIN is corrected during the trajectory of adaptation.

(**A**) Chromosome missegregation rates determined at monthly interval by live-cell imaging of parental RPE-1 p53KD cells and aneuploid clones at from Appendix Fig. S1A, divided into three subcategories: lagging chromosomes, anaphase bridges and others (multipolar spindle, polar chromosome, cytokinesis failure, binucleated cell). All conditions were analyzed blinded. Bars are averages of at least 2 experiments and a minimum of 50 cells were filmed per clone per experiment. Error bars indicate standard deviation. (**B**) Spearman correlation between the number of RNA-sequencing derived imbalanced and lost genes per clone and the level of CIN as percentage of total number of anaphases as determined in Fig. 4A. Dots represent mean, error bars indicate standard deviation, $n = 1$ or 2. Color-coding as determined in Fig. 2A.

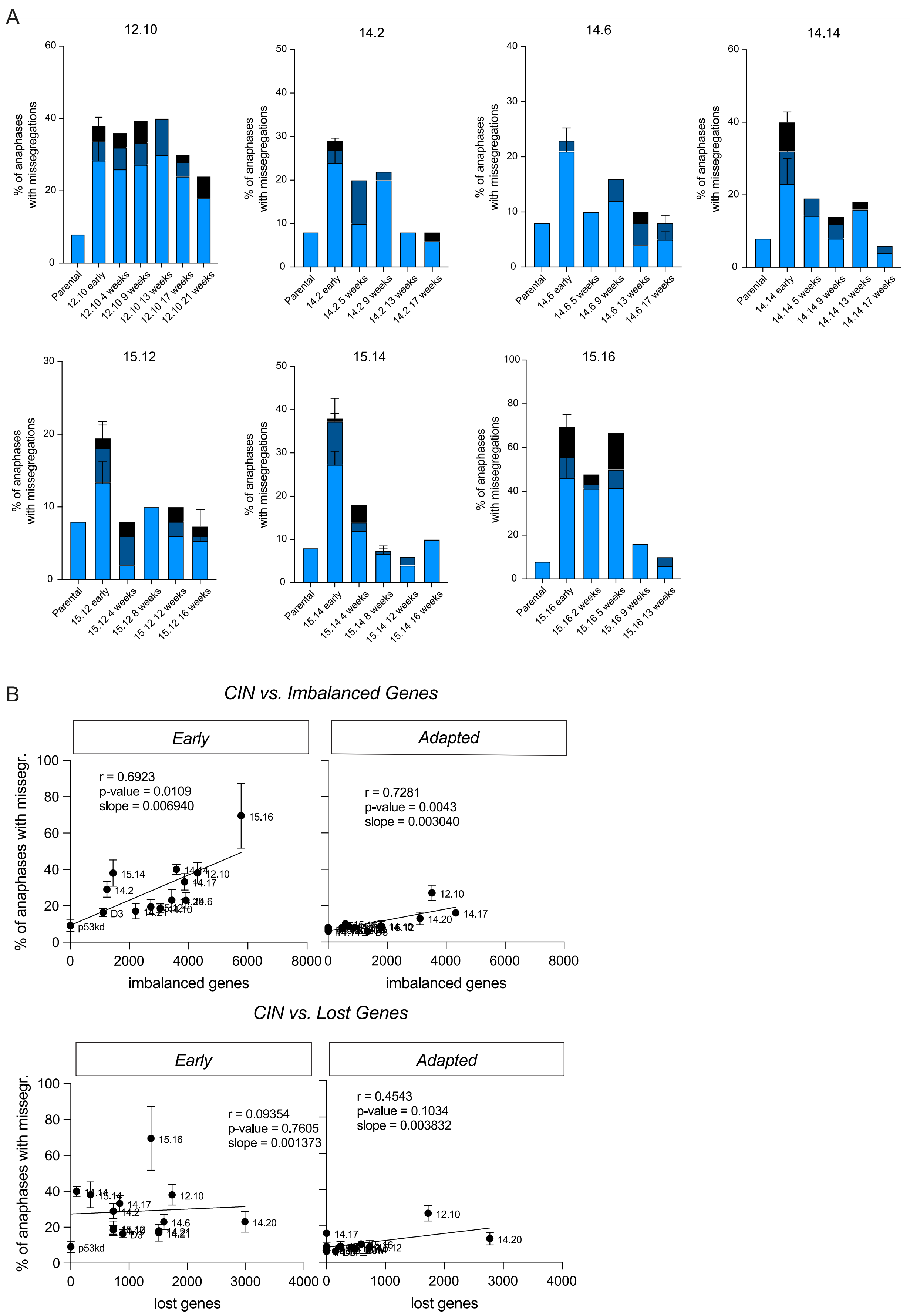
A
12.10
14.2
14.6
14.14
15.12
15.14
15.16
% of anaphases with missegregations
Parental
B
CIN vs. Imbalanced Genes
Early
Adapted
% of anaphases with missegr.
r = 0.6923
p-value = 0.0109
slope = 0.006940
r = 0.7281
p-value = 0.0043
slope = 0.003040
imbalanced genes
CIN vs. Lost Genes
r = 0.09354
p-value = 0.7605
slope = 0.001373
r = 0.4543
p-value = 0.1034
slope = 0.003832
lost genes

 © The Author(s)

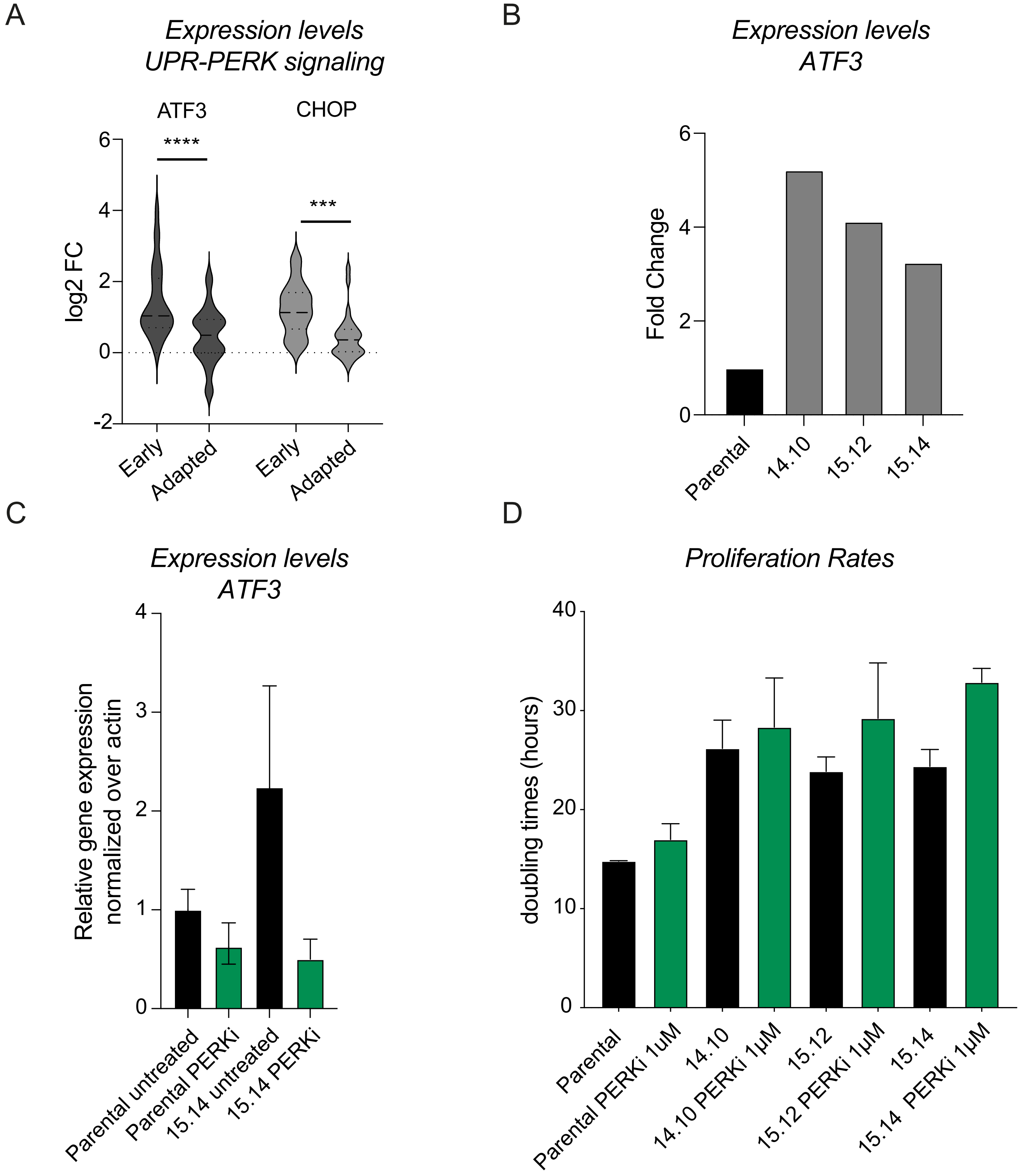
A
Expression levels
UPR-PERK signaling
ATF3
CHOP
****
***
log2 FC
6
4
2
0
-2
Early
Adapted
Early
Adapted
B
Expression levels
ATF3
Fold Change
6
4
2
0
Parental
14.10
15.12
15.14
C
Expression levels
ATF3
Relative gene expression
normalized over actin
4
3
2
1
0
Parental untreated
Parental PERKi
15.14 untreated
15.14 PERKi
D
Proliferation Rates
doubling times (hours)
40
30
20
10
0
Parental
Parental PERKi 1uM
14.10
14.10 PERKi 1µM
15.12
15.12 PERKi 1µM
15.14
15.14 PERKi 1µM

Figure EV2. Reduced PERK-signaling is not sufficient to drive adaptation.

(**A**) Violin plots showing the expression levels of ATF3 and CHOP in early and adapted clones extracted from the transcriptome data. An ordinary two-way ANOVA was performed between early and adapted clones ($n = 28$). P values are assigned according to GraphPad standard, P value for ATF3 = 0.000000002575531, P value for CHOP = 0.000001051675832. (**B**) Expression level of ATF3 of early clones normalized to parental cells extracted from transcriptome data for 3 individual clones. (**C**) mRNA levels of ATF3 determined via qRT-PCR of parental RPE-1 p53KD and clone 15.14 after 24 h of treatment with 1 μM of PERK inhibitor. Values were normalized to actin and are displayed relative to expression levels in untreated parental cells. Bars show mean expression levels of 3 technical replicates; error bars indicate upper and lower limits. (**D**) Average doubling times parental RPE-1 p53KD and selected early clones untreated and treated with 1 μM PERK inhibitor added 2 h prior to imaging, determined by live-cell imaging. Error bars indicate standard deviation, $n = 2$.

 © The Author(s)

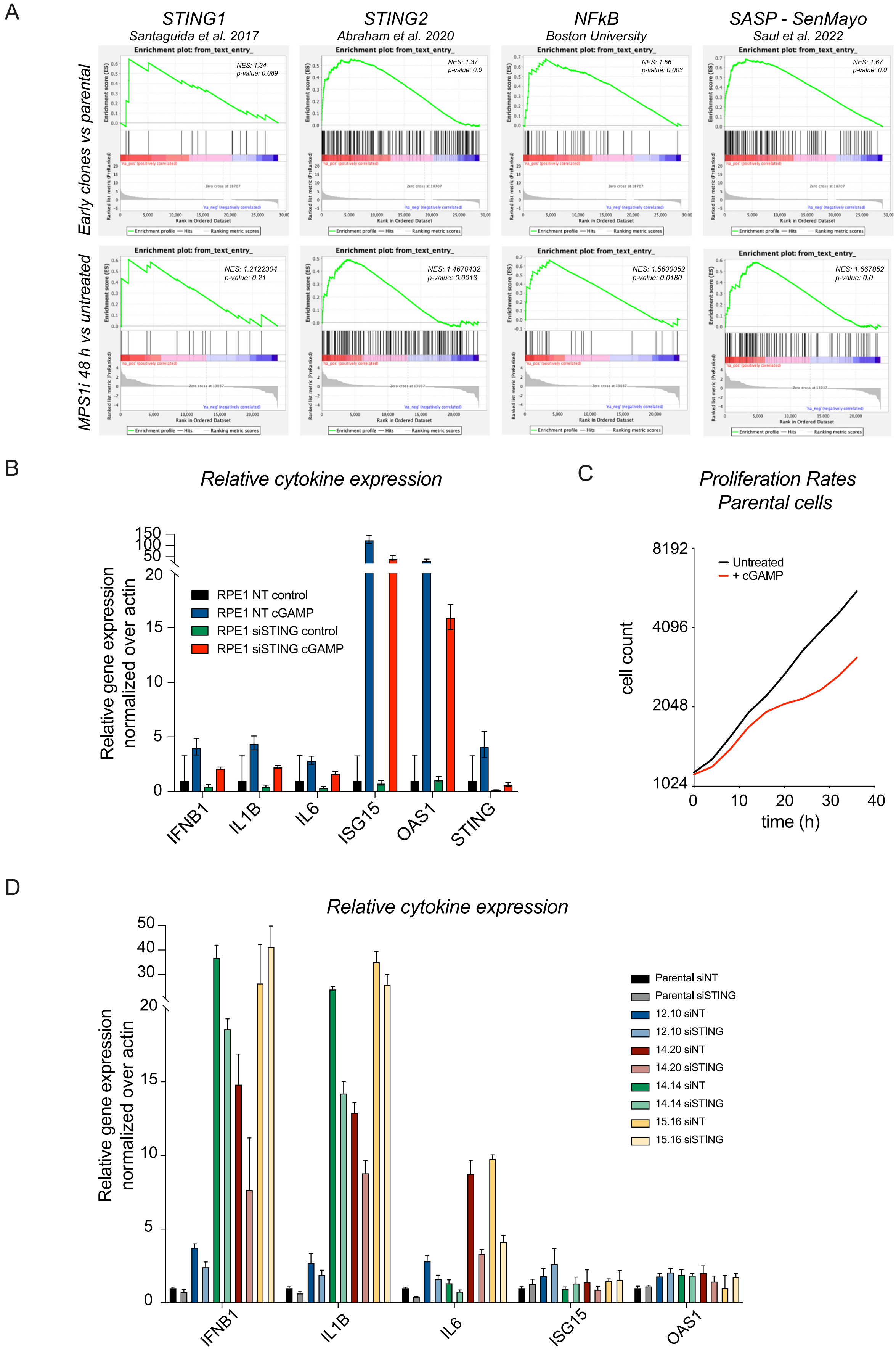
A
STING1
Santaguida et al. 2017
STING2
Abraham et al. 2020
NFkB
Boston University
SASP - SenMayo
Saul et al. 2022
Early clones vs parental
MPS1i 48 h vs untreated
Enrichment plot: from_text_entry_
NES: 1.34
p-value: 0.089
NES: 1.37
p-value: 0.0
NES: 1.56
p-value: 0.003
NES: 1.67
p-value: 0.0
NES: 1.2122304
p-value: 0.21
NES: 1.4670432
p-value: 0.0013
NES: 1.5600052
p-value: 0.0180
NES: 1.667852
p-value: 0.0
Enrichment score (ES)
Ranked list metric (PreRanked)
Rank in Ordered Dataset
Enrichment profile
Hits
Ranking metric scores
B
Relative cytokine expression
Relative gene expression
normalized over actin
RPE1 NT control
RPE1 NT cGAMP
RPE1 siSTING control
RPE1 siSTING cGAMP
IFNB1
IL1B
IL6
ISG15
OAS1
STING
C
Proliferation Rates
Parental cells
cell count
time (h)
Untreated
+ cGAMP
D
Relative cytokine expression
Relative gene expression
normalized over actin
Parental siNT
Parental siSTING
12.10 siNT
12.10 siSTING
14.20 siNT
14.20 siSTING
14.14 siNT
14.14 siSTING
15.16 siNT
15.16 siSTING
IFNB1
IL1B
IL6
ISG15
OAS1

Figure EV3. Characterization of the inflammatory response.

(**A**) Transcriptome data of early clones (top row) and of parental p53KD cells treated for 48 h with 50 nM CENP-Ei and 480 nM MPS1i tested against known STING signaling, NF-κB signaling and SASP signaling gene sets. An overview of the different gene sets can be found in Dataset EV1. A weighted Kolmogorov–Smirnov statistical test was used to determine enrichment scores. (**B**) mRNA levels of inflammatory response cytokines determined via qRT-PCR of parental RPE-1 p53KD after 72 h siRNA against STING, untreated or treated for 24 h with 67 μM of cGAMP. Values were normalized to actin and are displayed relative to expression levels in untreated parental cells. Bars show mean expression levels of 3 technical replicates; error bars indicate upper and lower limits. (**C**) Cell counts of parental RPE-1 p53KD cells left untreated or treated with 67 μM cGAMP which was added 2 h prior to imaging. Y-axis Is displayed in a Log2 scale. (**D**) mRNA levels of inflammatory response cytokines determined via qRT-PCR. RNA of parental RPE-1 p53KD and early clones were isolated 72 h post siRNA transfection with a NT siRNA or an siRNA against STING. Values were normalized to actin and are displayed relative to expression levels in parental cells treated with non-targeting siRNA. Bars show mean expression levels; error bars indicate upper and lower limits.

 © The Author(s)

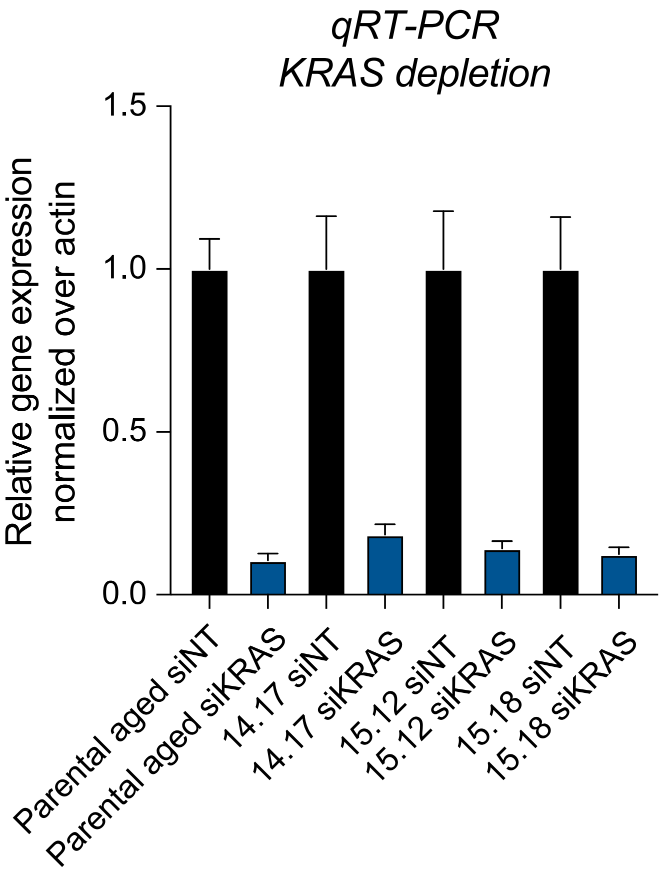

Figure EV4. KD of KRAS in adapted clones.

mRNA levels of KRAS determined via qRT-PCR of aged parental RPE-1 p53KD and adapted clones 24 h after siRNA transfection with a KRAS siRNA or a NT siRNA. Values were normalized to ribophorin and are displayed relative to expression levels in cells treated with siNT. Bars show mean expression levels of 3 technical replicates; error bars indicate upper and lower limits.

© The Author(s)
